# Segmental and global amyloid burden and myocardial mechanics: a multimodality imaging comparison

**DOI:** 10.25122/jml-2026-0071

**Published:** 2026-05

**Authors:** Claudiu Stan, Gabriela Neculae, Sebastian Onciul, Robert Daniel Adam, Laura Andrei, Razvan Capşa, Andreea Jercan, Sorina Badelita, Daniel Coriu, Ruxandra Jurcuţ

**Affiliations:** 1 Carol Davila University of Medicine and Pharmacy, Bucharest, Romania; 2 Department of Nuclear Medicine and Ultrasonography, Fundeni Clinical Institute, Bucharest, Romania; 3 Department of Cardiology, Expert Center for Rare Genetic Cardiovascular Diseases, Prof. Dr. C.C. Iliescu Emergency Institute for Cardiovascular Diseases, Bucharest, Romania; 4 Department of Cardiology, Floreasca Emergency Clinical Hospital, Bucharest, Romania; 5 Department of Radiology, Fundeni Clinical Institute, Bucharest, Romania; 6 Department of Hematology, Fundeni Clinical Institute, Bucharest, Romania

**Keywords:** ATTR-CM, cardiac amyloidosis, SPECT, cardiac MRI, semiquantitative uptake, ECV, GLS, 99mTc, technetium-99m, AL, amyloid light-chain, ATTR, transthyretin amyloidosis, ATTR-CM, transthyretin amyloid cardiomyopathy, BS, bone scintigraphy, CA, cardiac amyloidosis, cMRI, cardiac magnetic resonance imaging, DPD, 3,3-diphosphono-1, 2-propanodicarboxylic acid, ECG, electrocardiography, Glu54Gln, glutamic acid to glutamine substitution at position 54, GLS, global longitudinal strain, H/CL, heart-to-contralateral lung uptake ratio, HDP, hydroxymethylene diphosphonate/oxidronate (Technescan™ HDP), H/L, heart-to-liver uptake ratio, LV, left ventricle, LEHR, low-energy high-resolution, LVEF, left ventricular ejection fraction, NT-proBNP, N-terminal pro–B-type natriuretic peptide, PET, positron emission tomography, PYP, pyrophosphate (Technescan™ PYP), ROI, region of interest, SPECT, single-photon emission computed tomography, TnI, high-sensitivity troponin I, TTE, transthoracic echocardiography, TTR, transthyretin, and WB, whole body

## Abstract

Bone scintigraphy with bisphosphonate tracers is a cornerstone of noninvasive diagnosis in transthyretin amyloid cardiomyopathy (ATTR-CM). However, methods for estimating the extent and regional distribution of myocardial amyloid burden using single-photon emission computed tomography (SPECT) remain insufficiently validated. This study aimed to evaluate whether global and segmental myocardial radiotracer uptake, assessed by bisphosphonate SPECT, correlates with myocardial amyloid infiltration measured by cardiac magnetic resonance (cMRI)-derived extracellular volume (ECV), and to explore the relationship between these imaging markers and myocardial mechanics. Twenty-seven patients with ATTR-CM who underwent multimodality imaging with bisphosphonate SPECT, cMRI, and transthoracic echocardiography (TTE) between 2017 and 2025 were retrospectively screened. Sixteen patients with complete segmental datasets across all imaging modalities were included in the final analysis. Segmental mixed-effects models were used to account for clustering of myocardial segments within patients. Segmental extent of amyloid burden assessed by SPECT was significantly associated with segmental ECV (β = 0.042, SE = 0.010, t = 4.22), indicating higher radiotracer retention in segments with greater myocardial infiltration. Segmental ECV demonstrated a strong association with impaired longitudinal strain (β = 0.269, SE = 0.043, t = 6.22). In contrast, the direct relationship between radiotracer uptake and longitudinal strain was weaker and not statistically significant (β = 0.016, SE = 0.010, t = 1.60). In combined models, ECV remained independently associated with strain (β = 0.266, SE = 0.045, t = 5.96), whereas radiotracer uptake lost significance (β = 0.003, SE = 0.010, t = 0.29). At the global level, global extent amyloid burden (GEAB) correlated significantly with ECV (β = 0.435, *P* = 0.018), while the association between GEAB and global longitudinal strain was weaker and nonsignificant (β = 0.092, *P* = 0.10). Semiquantitative myocardial uptake on bisphosphonate SPECT demonstrates significant concordance with cMRI-derived extracellular volume, supporting its role as a surrogate marker of myocardial amyloid burden. However, myocardial mechanics appear to be more closely associated with extracellular expansion than with radiotracer uptake itself. These findings suggest that current SPECT techniques can estimate myocardial amyloid distribution but remain limited in segmental precision compared with cMRI, likely due to the spatial resolution of conventional SPECT systems and the lack of dedicated processing algorithms for cardiac amyloidosis.

## Introduction

Amyloidosis is a group of protein-misfolding diseases characterized by the extracellular deposition of insoluble fibrils, leading to organ dysfunction. In particular, 98% of cardiac amyloidosis cases are due to light chain (AL) and transthyretin (ATTR) amyloidosis [1]. AL amyloidosis is a clonal plasma cell disorder characterized by the formation of free light chains of immunoglobulins into an amorphous material deposited extracellularly in multiple organs and systems. ATTR amyloidosis occurs due to disaggregation of the transthyretin protein tetramer produced in the liver and the pathological folding of the monomers with the formation of oligomers and protein fibrils. Fibrillar transthyretin amyloid can be wild-type or age-related (ATTRwt) or due to mutant variants of the gene encoding transthyretin (ATTRv) [2,3].

The Glu54Gln mutation remains the most widespread variant in Romania, with an endemic prevalence in the northeastern area of Suceava County [4,5]. This variant is associated with a mixed phenotype of cardiac and neurological manifestations [5,6]. Previous studies have demonstrated the excellent sensitivity and specificity of bisphosphonate scintigraphy for detecting ATTR-CM associated with the Glu54Gln mutation [7].

The non-invasive diagnosis of ATTR-CM has already been implemented by international multi-societal imaging guidelines [8-11]. An early, reliable diagnosis is key to the appropriate initiation of therapy. It prevents the development of irreversible complications, thereby stopping the disease progression [12]. Monitoring of therapeutic response is an important clinical issue, assisted by the quantification of hemodynamic, biochemical, and morphological parameters. However, the specific role of each monitoring technique is disputed, and the quantification of amyloid deposition in the myocardium remains the domain of invasive methods [13]. In recent years, however, imaging quantification of extracellularly stored amorphous material at the cardiac level has replaced the need for cardiac biopsy [14]. Nuclear techniques such as bisphosphonate scintigraphy provide qualitative measures of myocardial involvement, including Perugini grades 0-3 and the H/CL ratio [8,10,12,15]. Also, myocardial amyloid burden can be quantified using cardiac MRI to assess the extracellular space [16].

The present study aimed to evaluate whether global and segmental radiotracer uptake, assessed by cardiac SPECT, correlates with myocardial amyloid infiltration, as measured by cMRI-derived ECV. In addition, we explored the relationship between these imaging parameters and myocardial mechanics assessed by longitudinal strain. We hypothesized that radiotracer uptake would demonstrate significant concordance with myocardial infiltration. The most important application of amyloid mapping could be the assessment of disease regression following therapy [17].

## Material and Methods

### Study population

A multicenter study was conducted between January 2017 and January 2025, involving two university centers and a specialized cardiac MRI center in Bucharest, Romania. The study included 27 patients diagnosed with transthyretin-associated cardiomyopathy (ATTR-CM) who underwent cardiac magnetic resonance imaging (cMRI) and bisphosphonate scintigraphy (BS). However, only 16 patients with complete segmental data from all imaging modalities were included in the final analysis. Among these patients, 11 carried the Glu54Gln mutation, one carried the Glu89Val mutation, and four were diagnosed with ATTRwt-CM.

All patients signed an informed consent form regarding the processing of personal data for clinical and research purposes. They agreed to the administration of bisphosphonates for diagnostic purposes in accordance with local Ethical Committee regulations. There were no contraindications to any imaging investigation. Pregnant or breastfeeding women were excluded from radionuclide investigations.

### Imaging acquisition and analysis

#### Standard transthoracic echocardiography (TTE)

TTE was performed according to the guidelines of the European Association for Cardiovascular Imaging. We used a Vivid ultrasound scanner (GE Vingmed Ultrasound, Horten, Norway). Left ventricular (LV) systolic function was assessed using Simpson’s method to calculate left ventricular ejection fraction (LVEF) in both 4- and 2-chamber views. Myocardial deformation was assessed by 2D speckle tracking at the segmental level and by calculating global longitudinal strain (GLS).

#### Cardiac Magnetic Resonance Imaging (cMRI)

CMR was performed on a 1.5-Tesla scanner (Siemens Magnetom Sempra) equipped with a dedicated cardiac phased-array coil, employing standard sequences to evaluate left ventricular end-diastolic volume and ejection fraction. Late gadolinium enhancement imaging was performed 10 minutes after intravenous injection of 0.15 mmol/kg gadobutrol. Native and post-contrast T1 mapping were acquired in basal, mid-, and apical short-axis views. Tempus Pixel post-processing software was used to calculate extracellular volume fraction (ECV). Segmental ECV values were derived according to the American Heart Association (AHA) 16-segment model.

#### Cardiac Bisphosphonates SPECT (cSPECT)

cSPECT was performed based on the consensus recommendations by Dorbala *et al*. [8-10] for multimodality imaging in cardiac amyloidosis. The radiolabeled bisphosphonate was administered intravenously. Most of the included patients (*n* = 15, 93%) received 99mTc-HDP (Oxidronate, Technescan™ HDP, Curium, Petten, The Netherlands) at an average dose of 700 MBq (±10%). One patient received the same dose but with 99mTc-PYP (Pyrophosphate, Technescan™ PYP, Curium, Petten, The Netherlands). Two types of gamma camera scanners were used: a SPECT system (Siemens e.cam, Dual Head Signature Series, 2007, Chicago, IL, USA) and a hybrid SPECT-CT system (GE Optima NM/CT 640, 2015, Haifa, Israel).

The planned acquisitions included whole-body (WB) images in both anterior and posterior views, as well as thoracic planar views. Semi-quantitative analysis comprised heart-to-contralateral lung (H/CL) and heart-to-liver (H/L) ratios, as well as visual Perugini grading. For SPECT imaging, a wide-field-of-view (FOV) with two active detectors was oriented at 90°. We selected 32 frames per detector, with a 180° rotation and 25 seconds per frame. The chosen matrix sizes were 64x64 for the Siemens scanner and 128x128 for the GE SPECT-CT system, with a zoom factor of 1.45. A low-energy, high-resolution (LEHR) collimator was used for the acquisitions.

#### Processing and interpretation of the results

After analyzing the raw SPECT images, we performed a three-axis reconstruction: short, long vertical, and long horizontal. The next step involved masking the radiotracer accumulation outside the left ventricle (LV) and selecting the LV center for normalization. We used the Cedars-Sinai QPS program for SPECT quantification (quantifying perfusion SPECT) and created bull’s-eye images based on the AHA-17 segments to analyze segmental radiotracer accumulation at rest. We chose the color code “Thermal” for polar maps and “Cool” for slices.

From the database, we selected either FemaleRestMB or MaleRestMB (based on sex, study conditions, whether stress or rest, and the radiotracer Sestamibi for myocardial perfusion), as there is no database for bisphosphonate SPECT in cardiac amyloidosis.

We calculated the global extent of amyloid burden (GEAB) as a percentage difference (100 - x), where x represents the uptake defect. A greater radiotracer uptake defect corresponds to a lower GEAB.

The polar map and bull’s-eye in the “Rest Extent” window of the bisphosphonate uptake defects allowed us to calculate amyloid infiltration for each segment separately. We obtained the segmental extent of amyloid burden (SEAB) as a percentage difference (100 - x), where x represents the uptake defect for each segment. Like GEAB, a larger defect results in a lower degree of segmental amyloid infiltration.

### Segmental analysis

Regional myocardial uptake, extracellular volume, and longitudinal strain were analyzed across the same 16 myocardial segments with image registration. Segmental datasets were merged to create a hierarchical structure in which myocardial segments were nested within patients.

Base-to-apex gradients were calculated to quantify regional heterogeneity of myocardial infiltration, tracer uptake, and mechanical function. For each patient, basal values were obtained by averaging measurements from segments 1–6, whereas apical values were derived from segments 13–16 according to the American Heart Association 16-segment model. The base-to-apex gradient (Δ) was defined as the difference between basal and apical values (Δ = basal − apical) for extracellular volume (ΔECV), scintigraphic uptake (Δuptake), and longitudinal strain (Δstrain).

### Statistical analysis

Continuous variables are presented as mean ± standard deviation or median with interquartile range, as appropriate, based on data distribution. Categorical variables are reported as counts and percentages.

Because multiple myocardial segments were analyzed within each patient, a hierarchical data structure was accounted for using linear mixed-effects models with the patient identifier included as a random intercept to address within-subject clustering. Segment-level analyses were performed to evaluate relationships between regional scintigraphic uptake, extracellular volume, and longitudinal strain.

Primary mixed-effects models were constructed to assess: (1) the association between regional scintigraphic uptake and segmental ECV, (2) the relationship between segmental ECV and longitudinal strain, and (3) the association between scintigraphic uptake and longitudinal strain. A combined multivariable mixed-effects model including both radiotracer uptake and ECV was used to explore the relationship between radiotracer uptake and myocardial mechanics.

To quantify regional heterogeneity, base-to-apex gradients were calculated at the patient level for uptake, ECV, and longitudinal strain. Basal values were derived from the average of segments 1–6, whereas apical values were obtained from segments 13–16 according to the American Heart Association 16-segment model. Gradients (Δ) were defined as basal minus apical values for each parameter. Associations between patient-level gradients were evaluated using Spearman correlation analysis.

Exploratory linear regression models were performed to assess determinants of global longitudinal strain (GLS), including global ECV and base-to-apex gradients as candidate predictors.

Model assumptions were evaluated by visual inspection of residual distributions. A two-sided *P* value <0.05 was considered statistically significant. Statistical analyses were performed using R software (R Foundation for Statistical Computing, Vienna, Austria).

## Results

### Patient population

Sixteen patients with ATTR cardiomyopathy were included, providing a total of 256 myocardial segments for analysis. The main characteristics of the 16 patients included in the study are presented by ATTR-CM type (variant or wild-type) in Table 1.

Main global imaging parameters are summarized in Table 2.

**Table 1:** Summary of symptomatic patients’ demographics and clinical features

Clinical characteristics	ATTRv-CM symptomatic patients’ data (*n* = 12)	ATTRwt-CM symptomatic patients’ data (*n* = 4)
Age at diagnosis (y); median [IQR]	45 [44-48]	83 [81,86]
Male/ATTRv, *n* (%)	5 (42)	4(100)
BMI (kg/m^2^) [IQR]	24.5 [22.5-26.9]	23 [20.8-24.4]
NT-proBNP (pg/ml) [IQR]	474 [53.8 – 1378.5]	6717.5 [3181-7789.5]
TnI (ng/ml) [IQR]	0.02 [0.008-0.033]	0.041 [0.017-0.12]
eGFR (ml/min)	105 [89-113]	49 [47.5-74.5]
Phenotype *n* (%) Predominantly cardiac Mixed (cardiac + neurologic)	- 12 (100%)	4 (100%) -
NYHA functional class *n* (%) No HF symptoms reported I II III IV	6 (50%) 2 (17%) 4 (33%) 0 0	0 0 3(75%) 1(25%) 0
Syncope, *n* (%)	2(17%)	0
ICD, *n* (%)	0	0
Pacemaker, *n* (%)	0	0
Autonomic involvement, *n* (%)	10 (83%)	2(50%)
Peripheral neuropathy, *n* (%)	10 (83%)	4(100%)
Bilateral CTS, *n* (%)	11 (92%)	3(75)

* values are *n* (%), or median [interquartile range]; BMI, body mass index; BSA, body surface area; CTS, carpal tunnel syndrome; HF, heart failure; NYHA, New York Heart Association; NT-proBNP, N-terminal (NT)-pro hormone B-type natriuretic peptides; TnI, troponin I; ICD, implantable cardioverter-defibrillator.

**Table 2:** Summary of global imaging parameters according to ATTR-CM type

Clinical characteristics	ATTRv-CM (*n* = 12)	ATTRwt-CM (*n* = 4)
Perugini grade, *n* (%) Grade III Grade II	9 (75) 3 (25)	3 (75) 1 (25)
H/CL ratio [IQR]	1.85 [1.72-2.05]	1.95 [1.69-2.21]
H/L ratio [IQR]	2.61 [2.32-2.77]	2.80 [2.07-3.46]
GEAB [IQR]	76 [68-84]	69 [56-79]
GECV [IQR]	50.2 [42.4-59]	45.1 [39-51.3]
Global T2 (ms) [IQR]	49.5 [48-51.9]	49.5 [46.9-51.9]
IVS (mm) [IQR]	13.5 [12.5-16.5]	15.5 [13.5-18.5]
PW (mm) [IQR]	13 [11-16]	11.5 [10-13.5]
LV-EDD (mm) [IQR]	44.5 [38-51]	47.5 [44-49.5]
LV-EDV (ml) [IQR]	80 [67.5-96.5]	86 [74.5-95]
LVMi (g/m2) [IQR]	137 [104.6-151.8]	139.3 [120-160.6]
GLS [IQR]	-14.9 [-16.8- -12.9]	-13.7 [-17.8 - -11.2]
LVEF [IQR]	58 [54.5-59.5]	62.5 [59-64]
LAVi [IQR]	31.9 [26.4-40.9]	43.5 [40.9-51.9]

* values are *n* (%), or median [interquartile range]; GEAB, global extent amyloid burden; GECV, global extracellular volume; GLS, global longitudinal strain; H/CL ratio, heart to contralateral radio; H/L, heart to liver radio; IVS, interventricular septum; LAVi, left atrial volume indexed; LVEF, left ventricular ejection fraction; LV-EDD, left ventricular end-diastolic diameter; LV-EDV, left ventricular end-diastolic volume; LVMi, left ventricular mass index; PW, posterior wall.

### Segmental relationships between scintigraphic uptake, extracellular volume, and myocardial mechanics

**Figure 1:**
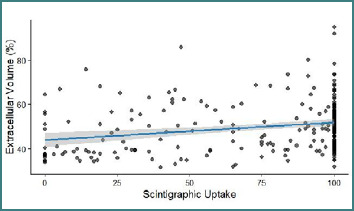
Relationship between scintigraphic uptake and extracellular volume. A scatter plot illustrates the association between segmental scintigraphic uptake and extracellular volume. Increased tracer uptake was associated with higher myocardial ECV, indicating greater amyloid infiltration.

Across myocardial segments, higher radiotracer uptake was significantly associated with increased extracellular volume. In mixed-effects analysis, regional radiotracer uptake independently predicted segmental ECV (β = 0.042, SE = 0.010, t = 4.22), indicating that segments with greater radiotracer retention demonstrated more extensive myocardial infiltration (Figure 1).

**Figure 2:**
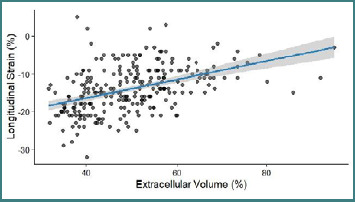
Relationship between regional extracellular volume and longitudinal strain. A scatter plot shows the association between segmental extracellular volume (ECV) and longitudinal strain across all myocardial segments. The solid line represents linear regression with 95% confidence intervals. Higher ECV values were associated with more impaired longitudinal strain.

Extracellular volume showed a strong association with myocardial mechanics. Higher segmental ECV values were significantly associated with greater longitudinal strain impairment (β = 0.269, SE = 0.043, t = 6.22), supporting a close relationship between myocardial infiltration and functional impairment (Figure 2).

The direct association between radiotracer uptake and longitudinal strain was weaker and did not reach statistical significance (β = 0.016, SE = 0.010, t = 1.60). However, when both bisphosphonate uptake and ECV were included in the same model, ECV remained independently associated with strain (β = 0.266, SE = 0.045, t = 5.96), whereas radiotracer uptake was no longer significant (β = 0.003, SE = 0.010, t = 0.29).

In segmental mixed-effects analysis, higher scintigraphic uptake was significantly associated with increased extracellular volume (β = 0.042, SE = 0.010, *P* < 0.001), indicating that regional radiotracer retention corresponded to greater myocardial infiltration. Extracellular volume demonstrated a strong association with myocardial mechanics, with higher ECV values associated with greater longitudinal strain impairment (β = 0.269, SE = 0.043, *P* < 0.001). In contrast, the direct association between scintigraphic uptake and longitudinal strain did not reach statistical significance (β = 0.016, SE = 0.010, *P* = 0.11).

When both radiotracer uptake and extracellular volume were included in the same model, extracellular volume remained independently associated with longitudinal strain (β = 0.266, SE = 0.045, *P* < 0.001), whereas scintigraphic uptake was no longer significant (β = 0.003, SE = 0.010, *P* = 0.77).

Although both scintigraphic uptake and extracellular volume appear to reflect myocardial amyloid burden, ECV demonstrated a closer relationship with myocardial mechanics, likely owing to its more direct quantification of extracellular remodeling and superior spatial resolution compared with conventional SPECT imaging (Table 3).

**Table 3:** Mixed-effects models evaluating relationships between scintigraphic uptake, extracellular volume, and longitudinal strain

Model	Predictor	Beta	SE	CI	t	*P*
ECV ~ Uptake	Uptake	0,042	0,01	0.022–0.061	4,22	<0.001
Strain ~ ECV	ECV	0,269	0,043	0.184–0.353	6,22	<0.001
Strain ~ Uptake	Uptake	0,016	0,01	-0.004–0.037	1,6	0.110
Strain ~ Uptake + ECV	Uptake	0,003	0,01	-0.017–0.022	0,29	0.772
Strain ~ Uptake + ECV	ECV	0,266	0,045	0.178–0.353	5,96	<0.001

### Base-to-apex gradient analysis

**Figure 3:**
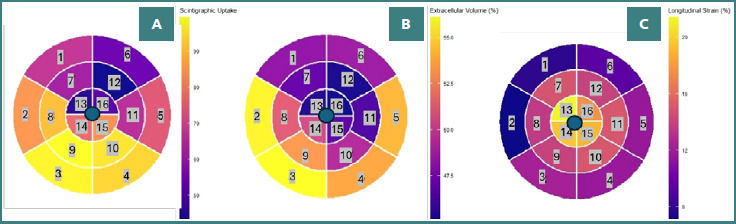
**Regional distribution of scintigraphic uptake, extracellular volume, and myocardial mechanics**. Polar maps illustrate: **A**, mean segmental values of scintigraphic uptake; **B**, extracellular volume; and **C**, longitudinal strain according to the 16-segment AHA model. A consistent base-to-apex gradient is observed across modalities, with higher uptake and extracellular volume and more impaired strain in basal segments.

Regional polar map analysis demonstrated a consistent base-to-apex distribution pattern across imaging modalities, with higher scintigraphic uptake, increased extracellular volume, and greater longitudinal strain impairment observed predominantly in basal myocardial segments (Figure 3A-C).

However, patient-level analysis of regional gradients did not demonstrate significant correlations between the infiltrative gradient (ΔECV) and the scintigraphic gradient (Δuptake) (ρ = −0.25, *P* = 0.36), nor between ΔECV and the mechanical gradient (Δstrain) (ρ = −0.13, *P* = 0.64).

These findings suggest that although a visually concordant regional distribution pattern is present across modalities, quantitative patient-level gradient relationships remain variable.

### Determinants of global function

In linear regression analysis evaluating determinants of global longitudinal strain, neither ΔECV nor Δuptake independently predicted GLS after adjustment for global ECV. The overall model was significant (R^2^ = 0.55, *P* = 0.02), although individual predictors did not reach statistical significance.

**Figure 4:**
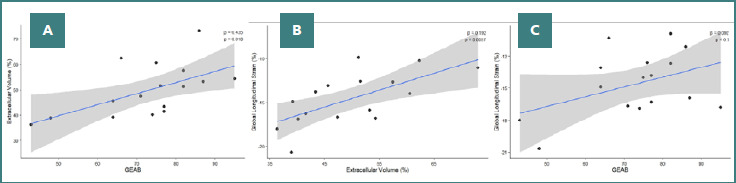
**Relationship between global extent amyloid burden (GEAB) on SPECT, ECV, and longitudinal function (GLS)**. **A**, GEAB is positively associated with ECV, reflecting higher radiotracer retention in patients with greater extracellular expansion. **B**, ECV demonstrates a significant association with GLS, with higher ECV values corresponding to impaired myocardial deformation. **C**, GEAB shows a weaker, non-significant association with GLS. Collectively, these findings suggest that the relationship between radiotracer uptake and myocardial function is mediated by the degree of extracellular expansion rather than a direct effect of tracer uptake.

In global analyses, global extent amyloid burden (GEAB) on scintigraphy demonstrated a significant positive association with extracellular volume (β = 0.435, *P* = 0.018), indicating higher tracer retention in patients with greater myocardial extracellular expansion. Extracellular volume, in turn, was significantly associated with global longitudinal strain (β = 0.192, *P* = 0.006), with higher ECV values corresponding to less negative strain, reflecting impaired myocardial deformation. In contrast, the association between GEAB and GLS was weaker and did not reach statistical significance (β = 0.092, *P* = 0.10) (Figure 4A-C).

### Case highlight

**Figure 5:**
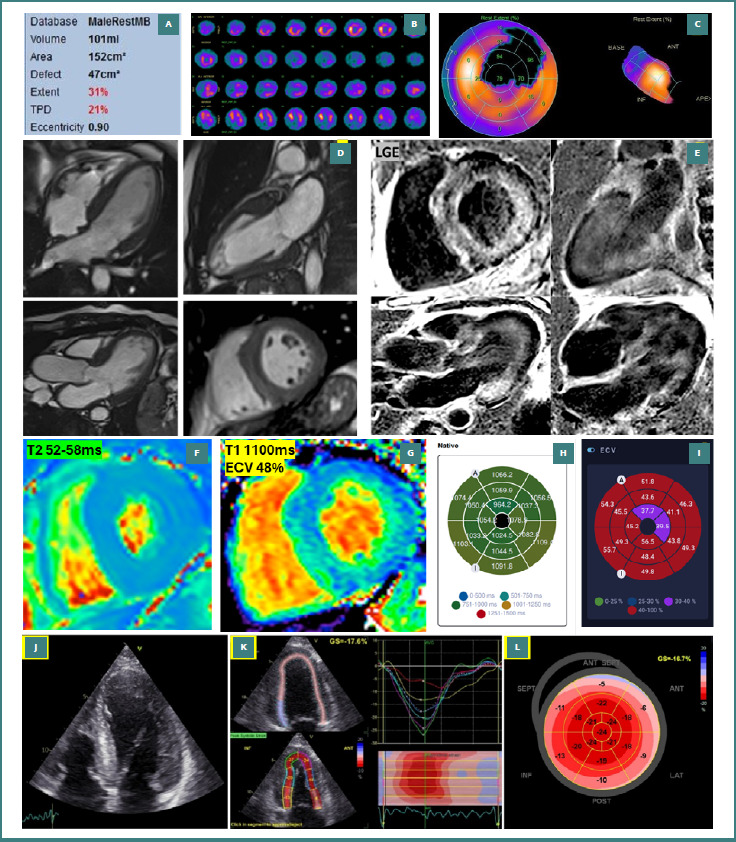
**A patient with good segmental correlation of amyloid distribution (SEAB), extracellular volume (ECV), and longitudinal strain (GLS). Cardiac bisphosphonate SPECT (A-C)**. The database shows appropriate limits for males in a resting state with Sestamibi (MB). Of the LV area, the 31% uptake defect indicates the amyloid-free region. **A**, The global amyloid burden (GEAB) is calculated as (100-31) = 69%, which represents the bisphosphonate uptake area. **B**, Slices in the short axis, from the base to the apex of the LV (first two rows), in the vertical long axis from the IVS to the lateral wall (row 3), and in the horizontal long axis from the anterior wall to the inferior wall (row 4). **C**, The bull's eye plot in AHA-17 segments estimates the percentage of uptake defect (x), from which the segmental amyloid burden extent, SEAB (100-x) %, is derived, along with a 3D extent defect of the LV. Cardiac MRI (D-I). **D**, The LV walls are thickened (IVS = 18 mm), the LV is nondilated, and the LVEF is 54%. The RV is nondilated, and the RVEF is 62%. The cardiac valves are supple. There is no pericardial fluid. **E**, Late gadolinium uptake (LGE) sequences reveal diffuse, circumferential, transmural contrast enhancement, but more intense at the subendocardial level, in the basal and middle segments of the LV, with relative apical sparing. We also note contrast enhancement in the atrial myocardium. **F**, A degree of diffuse, global edema is observed in the LV myocardium (T2 52–58 ms), and **G, H, I**, significant interstitial expansion with an increased extracellular volume fraction (ECV 48%). **J**, Transthoracic echocardiography demonstrating increased left ventricular wall thickness with a concentric hypertrophic phenotype. **K**, Speckle-tracking echocardiographic analysis illustrating reduced longitudinal deformation with segmental strain tracking and corresponding strain curves. **L**, Global longitudinal strain (GLS) bullseye polar map demonstrating the characteristic apical sparing pattern of cardiac amyloidosis, with relatively preserved apical strain and more impaired basal and mid-ventricular longitudinal deformation. ECV, extracellular volume; GLS, global longitudinal strain; IVS, interventricular septum; LGE, late gadolinium enhancement; LV, left ventricle; LVEF, left ventricular ejection fraction; MRI, magnetic resonance imaging; RVEF, right ventricular ejection fraction; RV, right ventricle; SPECT, single-photon emission computed tomography. Images from the author's collections.

Figure 5A-L shows a typical patient with ATTRGlu89Val-CM, with echocardiographic findings suggesting cardiac amyloidosis, showing good segmental correlation among amyloid distribution on SPECT (SEAB), extracardiac volume infiltration on cMRI (ECV), and longitudinal strain (GLS).

## Discussion

### Main findings

This study evaluated the relationship between semiquantitative bisphosphonate SPECT uptake, cMRI-derived extracellular volume, and myocardial mechanics in patients with transthyretin cardiac amyloidosis. The principal findings are:

1. Semiquantitative scintigraphic uptake demonstrated significant concordance with cMRI-derived extracellular volume at both global and segmental levels.
2. Both radiotracer uptake and extracellular volume appear to reflect myocardial amyloid burden.
3. Extracellular volume demonstrated a closer association with myocardial mechanics than scintigraphic uptake.
4. Segmental SPECT analysis identified regional amyloid distribution patterns similar to those observed by cMRI, although with lower spatial precision.

Collectively, these findings support the concept that semiquantitative cardiac SPECT may provide clinically meaningful estimates of myocardial amyloid distribution.

### Semiquantitative SPECT uptake as a marker of myocardial amyloid burden

Visual Perugini grading remains the cornerstone of noninvasive ATTR-CM diagnosis; however, interpretation may vary according to reader experience, particularly in borderline cases between grades 1 and 2. Quantitative tomographic approaches using SPECT or SPECT-CT have therefore emerged as potential tools for improving diagnostic reproducibility and estimating myocardial amyloid burden.

Previous studies using DPD- or HMDP-based SPECT-CT demonstrated significant correlations between quantitative uptake parameters such as SUVmax and Perugini grade, supporting the feasibility of tomographic quantification in ATTR cardiomyopathy [18–21]. In addition, variations in radiotracer uptake may be influenced by fibril composition and amyloid activity, suggesting that scintigraphic retention reflects not only extracellular expansion but also specific biological properties of transthyretin amyloid deposits [22-24].

In the present study, higher segmental radiotracer uptake was significantly associated with increased extracellular volume, indicating concordance between scintigraphic tracer retention and cMRI-derived myocardial infiltration. Similarly, global extent amyloid burden (GEAB) demonstrated a significant association with global ECV.

These findings support the concept that semiquantitative SPECT uptake reflects the presence and regional distribution of myocardial amyloid burden. However, compared with cMRI-derived ECV mapping, conventional SPECT imaging likely offers lower spatial precision due to the intrinsic spatial resolution of gamma-camera systems and the lack of dedicated processing algorithms optimized for cardiac amyloidosis imaging.

### Relationship between amyloid burden and myocardial mechanics

Extracellular volume and global longitudinal strain represent complementary domains of myocardial remodeling in ATTR cardiomyopathy. ECV quantifies extracellular expansion caused by amyloid deposition, whereas longitudinal strain reflects the functional consequences of myocardial infiltration. Consistent with previous studies, higher ECV values were strongly associated with impaired longitudinal deformation in our cohort [25-29]. The characteristic base-to-apex strain gradient with relative apical sparing further supports the established relationship between amyloid distribution and regional myocardial dysfunction.

In contrast, the direct association between segmental radiotracer uptake and longitudinal strain was weaker and did not remain significant after adjustment for extracellular volume. Although both scintigraphic uptake and ECV appear to reflect myocardial amyloid burden, cMRI-derived ECV demonstrated a closer association with myocardial mechanics.

This difference likely reflects the fact that ECV more directly quantifies the structural consequences of amyloid infiltration and provides superior spatial resolution compared with conventional SPECT imaging.

### Base-to-apex distribution patterns

Regional polar map analysis demonstrated a visually consistent base-to-apex distribution pattern across imaging modalities, with higher radiotracer uptake, increased extracellular volume, and greater longitudinal strain impairment predominantly observed in basal myocardial segments.

However, quantitative patient-level gradient analyses did not demonstrate significant correlations between ΔECV, Δuptake, and Δstrain. Similar observations have been reported in previous multimodality studies, suggesting that regional preservation of longitudinal deformation may depend not only on the proportion of amyloid infiltration but also on regional differences in total amyloid mass and myocardial remodeling [30].

### Clinical implications

Our findings suggest that semiquantitative SPECT analysis may provide clinically meaningful information regarding the extent and regional distribution of myocardial amyloid burden beyond conventional visual Perugini grading. As bisphosphonate scintigraphy with SPECT is a pivotal part of the diagnostic algorithm, developing dedicated software that can quantify uptake could provide a tool for predicting ECV, which is currently measured only by cMRI.

Potential applications include: monitoring disease progression, assessing response to anti-amyloid therapies, and improving multimodality risk stratification.

Nevertheless, cMRI-derived extracellular volume remains more closely associated with myocardial mechanics and may therefore better reflect the structural substrate of functional impairment.

### Limitations

This study has several limitations. First, the cohort size was relatively small because only a limited number of patients underwent complete multimodality imaging with SPECT, cMRI, and echocardiography. Second, the retrospective design may introduce selection bias; however, as each patient is their own control, this is less relevant.

Third, semiquantitative SPECT processing was performed using software originally developed for myocardial perfusion imaging rather than dedicated cardiac amyloidosis applications. Several technical factors may therefore influence the quantification of uptake, including attenuation artifacts, normalization methods, and the intrinsic spatial resolution of conventional gamma-camera systems.

These limitations likely contribute to the lower segmental precision of SPECT compared with cMRI-derived extracellular volume mapping.

### Future directions

Future studies should evaluate larger cohorts and develop dedicated cardiac amyloidosis SPECT processing software capable of standardized global and segmental quantification.

Longitudinal assessment of semiquantitative uptake parameters before and after disease-modifying therapy may help determine whether changes in scintigraphic amyloid burden parallel structural and functional remodeling assessed by cMRI and echocardiography.

In addition, improved spatial resolution with modern digital SPECT-CT systems may enhance the accuracy of regional amyloid burden estimation and strengthen multimodality correlations.

## Conclusion

Semiquantitative bisphosphonate SPECT demonstrates significant concordance with cMRI-derived extracellular volume and appears capable of estimating the global and regional distribution of myocardial amyloid burden in transthyretin cardiac amyloidosis.

Although both scintigraphic uptake and extracellular volume reflect myocardial amyloid burden, ECV demonstrated a closer association with myocardial mechanics, likely owing to its more direct quantification of extracellular remodeling and superior spatial resolution.

Further studies are required to validate standardized semiquantitative SPECT parameters and define their role in disease monitoring and therapeutic assessment in ATTR cardiomyopathy.

## References

[1] Buxbaum JN, Eisenberg DS, Fändrich M, McPhail ED, Merlini G, Saraiva MJM (2024). Amyloid nomenclature 2024: update, novel proteins, and recommendations by the International Society of Amyloidosis (ISA) Nomenclature Committee. Amyloid.

[2] Falk RH, Alexander KM, Liao R, Dorbala S (2016). AL (Light-Chain) Cardiac Amyloidosis: A Review of Diagnosis and Therapy. J Am Coll Cardiol.

[3] Ruberg FL, Berk JL (2012). Transthyretin (TTR) cardiac amyloidosis. Circulation.

[4] Hazenberg BPC, Bijzet J (2012). XIIIth International Symposium on Amyloidosis 2012: “From misfolded proteins to well-designed treatment” [Internet]. https://www.researchgate.net/publication/260086782_XIIIth_International_Symposium_on_Amyloidosis_2012_From_misfolded_proteins_to_well-designed_treatment.

[5] Jercan A, Ene A, Jurcut R, Draghici M, Badelita S, Dragomir M (2020). Clinical characteristics in patients with hereditary amyloidosis with Glu54Gln transthyretin identified in the Romanian population. Orphanet J Rare Dis.

[6] Neculae G, Zaroui A, Kharoubi M, Bézard M, Funalot B, Adam R (2025). A phenotypic comparison of the Romanian and French ATTRv cohorts: Glu54Gln founder pathogenic variant vs the most common variants in Western Europe. Int J Cardiol.

[7] Stan C, Neculae G, Adam RD, Jercan A, Badelita SN, Draghici MR (2025). Diagnostic Accuracy of Bisphosphonate Scintigraphy in Glu54GlnATTR Cardiomyopathy. J Clin Med.

[8] Dorbala S, Ando Y, Bokhari S, Dispenzieri A, Falk RH, Ferrari VA (2019). ASNC/AHA/ASE/EANM/HFSA/ISA/SCMR/SNMMI expert consensus recommendations for multimodality imaging in cardiac amyloidosis: Part 1 of 2-evidence base and standardized methods of imaging. J Nucl Cardiol.

[9] Dorbala S, Ando Y, Bokhari S, Dispenzieri A, Falk RH, Ferrari VA (2020). ASNC/AHA/ASE/EANM/HFSA/ISA/SCMR/SNMMI expert consensus recommendations for multimodality imaging in cardiac amyloidosis: Part 2 of 2-Diagnostic criteria and appropriate utilization. J Nucl Cardiol.

[10] Dorbala S, Ando Y, Bokhari S, Dispenzieri A, Falk RH, Ferrari VA (2022). Addendum to ASNC/AHA/ASE/EANM/HFSA/ISA/SCMR/SNMMI Expert Consensus Recommendations for Multimodality Imaging in Cardiac Amyloidosis: Part 1 of 2-Evidence Base and Standardized Methods of Imaging. J Card Fail.

[11] Garcia-Pavia P, Rapezzi C, Adler Y, Arad M, Basso C, Brucato A (2021). Diagnosis and treatment of cardiac amyloidosis: a position statement of the ESC Working Group on Myocardial and Pericardial Diseases. Eur Heart J.

[12] Writing Committee; Kittleson MM, Ambardekar AV, Cheng RK, Griffin JM, Maurer MS, Nativi-Nicolau J, Ruberg FL (2026). Transthyretin Cardiac Amyloidosis Evaluation and Management: 2025 ACC Concise Clinical Guidance. J Am Coll Cardiol.

[13] Grasso M, Cavaliere C, Vilardo V, Tagliani M, Di Toro A, Urtis M (2025). Present and future of endomyocardial biopsy in cardiac amyloidosis. Eur Heart J Suppl.

[14] Scully PR, Morris E, Patel KP, Treibel TA, Burniston M, Klotz E (2020). DPD Quantification in Cardiac Amyloidosis: A Novel Imaging Biomarker. JACC Cardiovasc Imaging.

[15] Perugini E, Guidalotti PL, Salvi F, Cooke RM, Pettinato C, Riva L (2005). Noninvasive etiologic diagnosis of cardiac amyloidosis using 99mTc-3,3-diphosphono-1,2-propanodicarboxylic acid scintigraphy. J Am Coll Cardiol.

[16] Haaf P, Garg P, Messroghli DR, Broadbent DA, Greenwood JP, Plein S (2016). Cardiac T1 Mapping and Extracellular Volume (ECV) in clinical practice: a comprehensive review. J Cardiovasc Magn Reson.

[17] Porcari A, Cuddy SA, Metra M, Emdin M, Fontana M, Gillmore JD (2026). How to monitor disease progression in ATTR amyloid cardiomyopathy: Implications for clinical practice and trial design. Eur J Intern Med.

[18] Ross JC, Hutt DF, Burniston M, Page J, Steeden JA, Gillmore JD (2018). Quantitation of 99mTc-DPD uptake in patients with transthyretin-related cardiac amyloidosis. Amyloid.

[19] Ramsay SC, Lindsay K, Fong W, Patford S, Younger J, Atherton J (2018). Tc-HDP quantitative SPECT/CT in transthyretin cardiac amyloid and the development of a reference interval for myocardial uptake in the non-affected population. Eur J Hybrid Imaging.

[20] Caobelli F, Braun M, Haaf P, Wild D, Zellweger MJ (2020). Quantitative 99mTc-DPD SPECT/CT in patients with suspected ATTR cardiac amyloidosis: Feasibility and correlation with visual scores. J Nucl Cardiol.

[21] Kessler L, Fragoso Costa P, Kersting D, Jentzen W, Weber M, Lüdike P, Carpinteiro A, Oubari S, Hagenacker T, Thimm A, Rassaf T, Herrmann K, Papathanasiou M, Rischpler C (2023). Quantitative 99mTc-DPD-SPECT/CT assessment of cardiac amyloidosis. J Nucl Cardiol.

[22] Ihse E, Rapezzi C, Merlini G, Benson MD, Ando Y, Suhr OB (2013). Amyloid fibrils containing fragmented ATTR may be the standard fibril composition in ATTR amyloidosis. Amyloid.

[23] Pilebro B, Suhr OB, Näslund U, Westermark P, Lindqvist P, Sundström T (2016). (99m)Tc-DPD uptake reflects amyloid fibril composition in hereditary transthyretin amyloidosis. Ups J Med Sci.

[24] Landreh M, Rising A, Presto J, Jörnvall H, Johansson J (2015). Specific chaperones and regulatory domains in control of amyloid formation. J Biol Chem.

[25] Morioka M, Takashio S, Nakashima N, Nishi M, Fujiyama A, Hirakawa K (2022). Correlation Between Cardiac Images, Biomarkers, and Amyloid Load in Wild-Type Transthyretin Amyloid Cardiomyopathy. J Am Heart Assoc.

[26] Martinez-Naharro A, Hawkins PN, Fontana M (2018). Cardiac amyloidosis. Clin Med (Lond).

[27] Phelan D, Collier P, Thavendiranathan P, Popović ZB, Hanna M, Plana JC (2012). Relative apical sparing of longitudinal strain using two-dimensional speckle-tracking echocardiography is both sensitive and specific for the diagnosis of cardiac amyloidosis. Heart.

[28] Chacko L, Martone R, Bandera F, Lane T, Martinez-Naharro A, Boldrini M (2020). Echocardiographic phenotype and prognosis in transthyretin cardiac amyloidosis. Eur Heart J.

[29] Minamisawa M, Claggett B, Adams D, Kristen AV, Merlini G, Slama MS (2019). Association of patisiran, an RNA interference therapeutic, with regional left ventricular myocardial strain in hereditary transthyretin amyloidosis: the APOLLO study. JAMA Cardiol.

[30] Bravo PE, Fujikura K, Kijewski MF, Jerosch-Herold M, Jacob S, El-Sady MS (2019). Relative Apical Sparing of Myocardial Longitudinal Strain Is Explained by Regional Differences in Total Amyloid Mass Rather Than the Proportion of Amyloid Deposits. JACC Cardiovasc Imaging.

